# *Rhizopus stolonifer* biomass catalytic transesterification capability: optimization of cultivation conditions

**DOI:** 10.1186/s12934-023-02141-y

**Published:** 2023-08-14

**Authors:** Nadeem I. Elhussieny, Heba A. El-Refai, Sayeda S. Mohamed, Yousseria M. Shetaia, Hala A. Amin, Gerd Klöck

**Affiliations:** 1 Department of Life Science and Chemistry, Constructor University, Campus Ring 1, 28759 Bremen, Germany; 2 Department of Chemistry of Natural and Microbial Products, National Research Centre, Cairo, 12622 Egypt; 3 Institute of Environmental Biology and Biotechnology, University of Applied Sciences, 28199 Bremen, Germany; 4 Department of Microbiology, Ain Shams University, Abbassia, Cairo, 11566 Egypt

**Keywords:** Rhizopus, Biodiesel, Transesterification, Triglycerides, Biocatalysis, Fungal biomass, Whole-cell

## Abstract

**Background:**

Using fungal biomass for biocatalysis is a potential solution for the expensive cost of the use o enzymes. Production of fungal biomass with effective activity requires optimizing the cultivation conditions.

**Results:**

*Rhizopus stolonifer* biomass was optimized for transesterification and hydrolysis of waste frying oil (WFO). Growth and biomass lipolytic activities of *R. stolonifer* improved under shaking conditions compared to static conditions, and 200 rpm was optimum. As biomass lipase and transesterification activities inducer, olive oil was superior to soybean, rapeseed, and waste frying oils. Biomass produced in culture media containing fishmeal as an N-source feedstock had higher lipolytic capabilities than corn-steep liquor and urea. Plackett Burman screening of 9 factors showed that pH (5–9), fishmeal (0.25–1.7%, w/v), and KH_2_PO_4_ (0.1–0.9%, w/v) were significant factors with the highest main effect estimates 11.46, 10.42, 14.90, respectively. These factors were selected for response surface methodology (RSM) optimization using central composite design (CCD). CCD models for growth, biomass lipase activity, and transesterification capability were significant. The optimum conditions for growth and lipid modification catalytic activities were pH 7.4, fishmeal (2.62%, w/v), and KH2PO4 (2.99%, w/v).

**Conclusion:**

Optimized culture conditions improved the whole cell transesterification capability of *Rhizopus stolonifer* biomass in terms of fatty acid methyl ester (FAME) concentration by 67.65% to a final FAME concentration of 85.5%, w/w.

**Supplementary Information:**

The online version contains supplementary material available at 10.1186/s12934-023-02141-y.

## Background

Transesterification of vegetable oils to fatty acid alkyl esters (FAAE) provides biodiesel and feedstock for the synthesis of several chemicals that are of industrial importance. Alkanol-amides are used as nonionic surfactants, emulsifiers, thickeners, and plastifiers [1]. Fatty alcohols are essential pharmaceutical and cosmetics additives and lubricants [2]. Some isopropyl esters are pharmaceutical emollients [3]. FAAE is also applied to manufacture carbohydrate fatty acid esters used as nonionic surfactants or edible non-calorific oils [4].

Growing demand for optimal transesterification catalysts motivates research in this field. Generally, transesterification catalysts are classified into homogenous catalysts, miscible with the reactants, and heterogenous catalysts, immiscible with the reactants. The primary homogenous catalysts are chemicals, including alkaline catalysts such as NaOH and KOH and acid catalysts such as HCl and H_2_SO_4_. On the other hand, heterogeneous catalysts include chemical catalysts, acid, base, and bifunctional catalysts, in addition to enzymatic catalysts [5]. Recently, heterogeneous catalysts using the functional groups displayed on the surface of polymers, such as graphene, have been investigated [6].

Unlike enzymatic triglyceride transesterification, chemical catalysis consumes energy and increases the risk of undesirable by-products (soap and pigments) formation, which might lead to the inability to separate the FAAE from other reaction products [7]. The enzyme-catalyzed process is superior to the conventional chemical process in several aspects, including thermal stability, pH range, ease of handling, reusability, and the probability of continuous operation [8–10].

Lately, the use of biomass for the catalysis of biodiesel production by transesterification has attracted the interest of researchers. The use of biomass as a heterogeneous catalyst has been investigated [11]. The reported use of biomass in transesterification catalysis includes the use of carbonized biomass [12], immobilization of enzymes [13, 14], and the use of whole-cell catalysis [15] Whole-cell biocatalysis provides all the advantages of enzymatic catalysis, including mild reaction conditions and the capability to catalyze oils with high water and free fatty acids. In addition, using whole-cell catalysts reduces the cost of enzyme purification and provides a protective environment for the lipases displayed by the biomass.

Adapted fungal biomass for the catalysis of triglyceride transesterification has been reported [16]. Several members of the Eumycota were tested for biomass catalytic capability, mainly members of *Ascomycota* and *Zygomycota* [17–19]. Mucorelean fungi were among the early investigated fungi for transesterifying triglycerides, especially genera *Mucor* and *Rhizopus*.

The effect of the cultivation conditions on the fungal biomass transesterification of vegetable oils has been studied by several members of Mucorales [20, 21]. Physical factors such as fermentation temperature, initial pH, and agitation rate were critical for producing whole-cell lipase catalysts. Nutrients and their concentration in the culture media were also essential, especially C-source, N-source, minerals, and C/N ratio [22–24]. An efficient technique for optimizing the cultivation conditions involves screening design such as Plackett Burman followed by response surface methodology such as central composite design (CCD). One factor at a time (OFAT) is usually involved in resolving the optimization of categorical factors and testing wide ranges [25].

The optimization of the submerged cultivation conditions of *R. stolonifer* to maximize the catalytic capability of the produced fungal thalli has not been reported. Hence, in this study, OFAT and statistical screening and optimization designs were used to maximize the lipid catalytic activity of the biomass of *Rhizopus stolonifer* for waste cooking oil hydrolysis and transesterification. Understanding the studied factors' influence and interactions on the investigated responses was also evaluated.

## Materials and methods

### Fungal strain and inoculum preparation

In a previous study*, Rhizopus stolonifer* 1aNRC11 (MN689079) mutant G was isolated and mutated [17]. The mutant was preserved on potato dextrose agar (PDA) slants supplemented with olive oil, 1%. The inoculum was prepared by subculturing the mutant on PDA plates incubated at 28 °C for three days. Agar disks (5 mm diameter) were used as inoculum.

### Culture media and feedstock screening

Glucose-Polypeptone medium (glucose 20 gL^−1^, polypeptone 20 gL^−1^, KH_2_PO_4_ 5 gL^−1^, MgSO_4_.7.H_2_O 0.5 gL^−1^) supplemented with olive oil (30 mLL^−1^) was used as the primary medium for the submerged cultivation of *R. stolonifer* 1aNRC11 and assessment of its biomass transesterification capabilities. Corn steep liquor (CSL), urea, and fishmeal (N content: 7.04%, Moisture content: 8.1%) were tested as replacements for polypeptone. Inoculated cultures were incubated in a shaking incubator at 28 °C and 150 rpm for three days. Harvested biomass was collected by filtration using Whatman filter paper no. 1. followed by lyophilization using a freeze dryer (Martin Christ, alpha LSC basic; Germany).

### Investigation of agitation rate

Triplicates of Glucose-polypeptone 100 mL inoculated cultures in 250 mL Erlenmeyer flasks were incubated in shaking incubators with the same orbital diameter at 28 °C and different agitation rates (0, 100, and 200 rpm) for three days. Biomass was harvested and processed, as previously mentioned, to measure biomass lipolytic and transesterification activities.

### Screening for transesterification activity inducers

Glucose-Polypeptone media were prepared using different activity inducers, including olive, soybean, and rapeseed oils (3%, w/v). Inoculated cultures were incubated in an orbital shaker at 150 rpm and 28 °C for three days. Harvested biomass was processed, and the activities were determined.

### Statistical screening

Plackett Burman design was used for screening experiments of 9 factors (Table 1) in triplicates. The screening experiments were carried out in 100 mL conical flasks containing 50 mL culture medium. A five days old culture of *Rhizopus stolonifer* 1aNRC11 was used as the source of the inocula. Flasks were incubated in a shaking incubator at 200 rpm. Biomass was processed, and the lipolytic and transesterification activities of the biomass were determined.

**Table 1:** Plackett Burman design factors and levels

Factor	Symbol	Levels	
		− 1	1
Temperature (°C)	X_1_	25.00	30.00
pH	X_2_	5.00	9.00
Incubation period (days)	X_3_	2.00	4.00
Inoculum size (disk)	X_4_	2.00	4.00
Glucose concentration (%, w/v)	X_5_	0.50	3.50
Fishmeal concentration (%, w/v)	X_6_	0.25	1.75
KH_2_PO_4_ concentration (%, w/v)	X_7_	0.10	0.90
MgSO_4_.7H_2_O concentration (%, w/v)	X_8_	0.01	0.09
Olive oil concentration (%, w/v)	X_9_	1.00	3.00

### Response surface methodology (RSM)

pH, fishmeal, and potassium dihydrogen phosphate concentrations were optimized using a central composite design (CCD). The investigated levels of selected factors are listed in Table 2. Culture media were prepared using 0.9 g L^−^1 MgSO_4_ and 2 mLL^−1^ olive oil with different levels of investigated factors and their relative interactions as the design suggested in triplicates. Cultures were inoculated with three disks 5 mm in diameter and were incubated at 28 °C and 200 rpm for four days. The processed biomass was used to assess the transesterification activity.

**Table 2:** Experimental levels of the selected factors in CCD

Symbol	Factor	Levels		
		− 1	0	1
X_2_	pH	7.00	8.00	9.00
X_6_	Fishmeal concentration (%, w/v)	2.00	2.50	3.00
X_7_	KH_2_PO_4_ concentration (%, w/v)	1.00	1.25	1.50

### Lipase assay

Lyophilized biomass (0.5 g) was inoculated to an emulsion of 5.5 g WFO and 30 mL of 1 M Tris buffer, pH 7.5. The reaction was carried out in an orbital shaker at 35 °C and 200 rpm for two h. The reaction mixture was centrifuged for 10 min at 5000 rpm. A gram of supernatant, two drops of phenolphthalein color indicator, and 25 mL diethyl ether and ethanol (1:1) solvent mixture were titrated against freshly prepared 0.1 N NaOH in 100 mL Erlenmeyer flask. Lipase activity (as the amount of enzyme required to produce 1 μmol free fatty acid per min) was determined in unit per gram cell weight (U/g) [26].

### Transesterification reaction

Fractured lyophilized biomass, 0.5 g, was used as the biocatalyst for an emulsion of 5 g WFO and 0.75 mL of 1 M phosphate buffer pH 7.5 in a 50 mL Erlenmeyer flask. The reaction was carried out at 35 °C and 250 rpm for 72 h, while methanol doses were added at 0, 24, and 48 h to a final concentration of 3 M methanol. The reaction mixture was transferred to a 15 mL centrifuge tube and spun at 10,000 rpm for 5 min.

Fatty acid methyl ester (FAME) was detected in the supernatant by thin-layer chromatography (TLC) with silica gel 60 F254 (E. Merck, Mumbai, India) using a solvent system of hexane/diethyl ether/acetic acid. Spots were stained in an iodine chamber and were investigated by Just TLC software (Sweday, Lund, Sweden).

### FAME analysis

Agilent Technologies 6890N gas chromatography (GC) provided a flame ionization detector, and a capillary column (HP-5 5% phenyl methyl siloxane, 30 m by 320 μm by 0.25 mm) was used to quantify the FAMEs content. Peaks determination was carried out by comparing the retention time of FAMEs of the sample (100 mg) and a known concentration of FAME standard mixture C8–C24 (Sigma-Aldrich Chemical Co. St. Louis, MO, USA), each dissolved in 1 mL hexane. One μL sample was injected into the GC, where the oven was adjusted at 210 ℃, isothermally for 15 min, and helium was used as the carrier gas. The peak area was used to calculate the concentration of each FAME type, and the sum of the concentration was referred to as FAME concentration (%, w/w). The transesterification activity was expressed using FAME concentration (%, w/w).

### Statistical analysis

The data obtained were subjected to statistical analysis using IBM SPSS Statistics (Version 16. IBM, Chicago, USA). Statistical optimization was designed and analyzed using JMP statistical software (Version 8. SAS Institute Inc., Cary, NC).

## Results

### OFAT investigations

#### Agitation rate

A significant improvement in the growth and biomass capability to catalyze triglycerides hydrolysis and transesterification was observed by comparing the static fermentation against the shaking flasks fermentation (Table 3). Biomass produced under static conditions showed aerial growth and a fungal mat, while biomass grown in shaking flasks showed pellety growth that varies in size and hairiness. The increased agitation rate increased biomass concentration to 11.35 gL^−1^, lipase activity to 37.5 Ug^−1^, and the transesterification capability in terms of FAME conversion up to 51.78%.

**Table 3:** Means comparison of *Rhizopus stolonifer* 1aNRC11 biomass production, lipase, and transesterification capability at different agitation rates

Agitation rate (rpm)	Biomass (g/L)	Lipase (U/g)	FAME (%)
0	3.60c	7.86c	16.42c
100	8.43b	24.02b	38.46b
200	11.35a	37.50a	50.78a
300	11.59a	36.85a	51.02a

Means followed by the same letter are not significantly different at a 95% confidence level

#### Different oils

Different oils were examined to improve the catalytic lipid modification capability of *R. stolonifer* 1aNRC11 biomass. The culture medium was supplemented with 3% w/v of olive, soybean, rapeseed, and waste frying oil separately. The biomass concentration was significantly less in the culture medium containing rapeseed oil, while no significant differences were observed among the other tested inducers (Table 4). Insignificant differences in the triglycerides hydrolysis and transesterification capability of biomass produced in culture media containing rapeseed and waste frying oil were observed. Olive oil was selected for further investigations because of the observation that biomass produced in olive oil containing medium had significantly higher lipid modification capabilities.

**Table 4:** Means comparison of *Rhizopus stolonifer* 1aNRC11 growth and lipase and transesterification activities cultivated using different oils as inducers

Oil	Biomass (g/L)	Lipase (U/g)	FAME (%)
Olive oil	10.35a	36.88a	51.00a
Soybean oil	10.18a	28.89c	39.66d
Rapeseed oil	8.11c	32.45b	44.56c
Waste cooking oil	10.34a	33.24b	46.43b

Means followed by the same letter are not significantly different at a 95% confidence level

#### Replacing N source feedstock

Different N sources were tested, and the biomass concentration, lipase activity, and transesterification capability were measured (Table 5). The presence of urea in the culture media inhibited the growth of *R. stolonifer* 1aNRC11; hence the lipase activity and transesterification capability were not measured. The biomass concentration range between 10.33 and 11.18 gL^−1^, with no significant difference between polypeptone and fishmeal. Biomass produced in the medium contained CSL showed relatively low triglycerides hydrolysis and transesterification capabilities, 20.07 U/g and 25.73 FAME %, respectively. The biomass concentration, lipase activity, and transesterification capability values show no significant differences for the biomass produced in media containing fishmeal and polypeptone.

**Table 5:** Means comparison of *Rhizopus stolonifer 1aNRC11* growth and lipase and transesterification activities cultivated using different N sources

N-Source	Biomass (gL^−1^)	Lipase activity (Ug^−1^)	FAME (%)
Polypeptone	11.07a	41.56a	51.88a
Urea	00.00c	–	–
CSL	10.33b	20.07b	25.73b
Fishmeal	11.18a	42.00a	52.03a

Means followed by the same letter are not significantly different at a 95% confidence level

### Statistical screening using Plackett Burman design

Nine factors were investigated using the Plackett Burmann design. Transesterification catalytic capability in terms of FAME conversion percentage, lipase activity, and growth in terms of final biomass concentration were observed as responses. The design matrix selected for screening significant factors is shown in Table 6. The model adequacy was calculated. Influences of the investigated factors and statistical significance were filtered via main effect estimates, Student’s t-test, and P-values for analysis of variance (Table 7). Factors with confidence greater than 90% (Prob. > │t│ 0.1) were considered to affect the response significantly. The lowest P-value indicates the most significant factors on the investigated response.

**Table 6:** Plackett Burman Design matrix and experimental results

Runs	X_1_	X_2_	X_3_	X_4_	X_5_	X_6_	X_7_	X_8_	X_9_	FAME conc (%), replicates			Lipase activity (U/g), replicates			Biomass (gL^−1^), replicates		
										**1**	**2**	**3**	**1**	**2**	**3**	**1**	**2**	**3**
**01–03**	− 1	− 1	− 1	1	− 1	− 1	1	− 1	1	30.46	32.53	30.07	29.58	24.38	25.00	2.59	2.48	2.53
**04–06**	− 1	− 1	1	− 1	− 1	1	− 1	1	1	36.96	33.78	32.59	99.79	80.21	82.71	19.42	20.05	20.24
**07–09**	− 1	− 1	1	− 1	1	1	1	− 1	− 1	28.67	26.99	26.85	45.42	41.25	48.75	8.66	8.38	7.57
**10–12**	− 1	1	− 1	− 1	1	− 1	1	1	1	41.43	41.21	40.58	28.33	33.33	26.04	2.23	1.83	2.61
**13–15**	− 1	1	− 1	1	1	1	− 1	− 1	− 1	25.94	25.78	26.19	17.50	20.83	15.83	11.09	8.33	12.68
**16–18**	− 1	1	1	1	− 1	− 1	− 1	1	− 1	30.32	30.70	30.23	43.13	78.33	63.75	5.55	4.87	6.32
**19–21**	0	0	0	0	0	0	0	0	0	53.24	51.60	52.81	42.29	33.13	48.33	11.20	9.62	10.14
**22–24**	1	− 1	− 1	− 1	1	− 1	− 1	1	− 1	0.00	0.00	0.00	5.42	6.04	3.75	2.19	2.44	2.55
**25–27**	1	− 1	− 1	1	− 1	1	1	1	− 1	39.31	38.23	38.00	78.33	97.71	88.75	13.82	13.60	14.52
**28–30**	1	− 1	1	1	1	− 1	− 1	− 1	1	0.00	0.00	0.00	3.54	4.38	4.58	5.07	4.39	5.09
**31–33**	1	1	− 1	− 1	− 1	1	− 1	− 1	1	30.50	30.36	30.11	37.29	43.54	44.79	10.28	10.76	10.66
**34–36**	1	1	1	− 1	− 1	− 1	1	− 1	− 1	31.86	32.27	32.12	46.04	32.92	33.33	6.21	6.16	5.57
**37–39**	1	1	1	1	1	1	1	1	1	40.33	40.35	40.40	30.83	28.33	28.75	19.47	21.26	20.33

**Table 7:** Plackett Burman screening main effect estimates, Student’s t-test, and P-values for analysis of variance

Factors	X_1_	X_2_	X_3_	X_4_	X_5_	X_6_	X_7_	X_8_	X_9_
FAME									
Main effect	− 8.19	11.46	− 0.35	0.14	− 10.32	10.42	14.90	6.32	3.79
Prob > t	0.0039	< 0.0001	0.8946	0.9569	0.0005	0.0004	< 0.0001	0.0220	0.1575
P-value	< 0.0001	< 0.0001	0.1718	0.5974	< 0.0001	< 0.0001	< 0.0001	< 0.0001	< 0.0001
Lipase									
Main effect	− 10.32	− 6.48	9.42	− 3.08	− 35.37	24.37	6.20	21.37	− 6.20
Prob > t	0.0068	0.0776	0.0126	0.3920	< .0001	< .0001	0.0906	< .0001	0.0905
P-value	0.0018	0.0196	0.0026	0.2251	< .0001	< .0001	0.0238	< .0001	0.0238
Biomass									
Main effect	1.50	0.59	3.75	1.46	− 1.64	10.03	− 0.12	3.60	2.26
Prob > t	0.0254	0.3611	< 0.0001	0.0294	0.0154	< 0.0001	0.8519	< 0.0001	0.0013
P-value	0.0001	0.0591	< 0.0001	0.0002	< 0.0001	< 0.0001	0.7053	< 0.0001	< 0.0001

Evaluating the screening model based on the growth as a response reveals that initial pH and KH_2_PO_4_ concentration were insignificant; the most influential factors were fishmeal concentration, incubation period, and MgSO_4_.7H_2_O concentration, respectively. Only inoculum size was found insignificant for lipase activity as the response. Glucose concentration had the highest negative impact, and fishmeal and MgSO_4_.7H_2_O concentrations had the most substantial positive influence on lipase activity.

The biomass transesterification capability of the produced biomass is the primary response of interest in this investigation. For this response, two factors were found insignificant: Inoculum size and incubation period. On the other hand, glucose concentration among the seven significant factors was the only factor with a negative main effect. The main effect values of pH, Fimeal concentration, and KH_2_PO_4_ concentration were the highest among significant factors.

### Statistical optimization using response surface methodology (RSM)

Based on the statistical screening results of transesterification capability as a response, some factors were fixed: X_1_ = 25 °C, X_3_ = 3 days, X_4_ = 3 disks, X_5_ = 0.5%, X_8_ = 0.09%, and X_9_ = 3%. The response surface methodology (RSM) was adopted to optimize pH, Fimeal concentration, and KH_2_PO_4_ concentration using a central composite design (CCD). The design matrix, the corresponding results, and the levels of the investigated factors of RSM experiments to determine the effects of the three investigated factors are shown in Table 8.

**Table 8:** Central Composite Design matrix and experimental results

Runs	X_2_^*^	X_6_^**^	X_7_^***^	FAME conc (%), replicates			Lipase activity (IU/g), replicates			Freeze-dried biomass (gL^−1^), replicates		
				1	2	3	1	2	3	1	2	3
**01–03**	− 1	− 1	− 1	42.99	38.69	40.89	15.59	16.49	15.07	13.79	11.78	12.79
**04–06**	+ 1	− 1	− 1	50.85	48.95	51.75	19.44	21.51	20.38	26.16	24.15	25.15
**07–09**	0	− 1	0	45.23	41.03	43.13	16.69	17.63	16.16	18.75	16.74	17.74
**10–12**	− 1	− 1	+ 1	38.32	32.02	36.22	13.31	13.22	12.78	12.62	10.61	11.61
**13–15**	+ 1	− 1	+ 1	40.81	45.01	43.96	14.53	13.58	16.57	19.55	20.51	19.75
**16–18**	0	0	− 1	42.14	45.29	43.19	15.18	17.72	16.19	20.32	22.31	21.31
**19–21**	− 1	0	0	48.38	45.08	46.28	18.23	16.96	17.71	20.87	21.86	19.87
**22–24**	0	0	0	47.86	49.01	46.71	17.98	18.54	17.91	21.25	22.24	23.25
**25–27**	+ 1	0	0	53.04	48.84	49.89	20.51	21.46	19.47	24.29	25.28	23.29
**28–30**	0	0	+ 1	51.88	52.73	53.78	19.95	20.36	21.38	24.20	22.19	23.20
**31–33**	− 1	+ 1	− 1	52.57	52.72	49.42	20.29	21.36	19.24	18.71	18.70	19.71
**34–36**	+ 1	+ 1	− 1	50.76	49.96	48.66	19.40	21.00	18.87	22.50	20.95	21.92
**37–39**	0	+ 1	0	61.26	58.11	59.16	24.53	25.99	24.01	25.50	23.49	24.49
**40–42**	− 1	+ 1	+ 1	75.70	69.40	68.30	31.60	31.52	28.48	31.81	29.80	30.80
**43–45**	+ 1	+ 1	+ 1	72.70	68.40	70.60	30.14	31.03	29.61	26.49	24.48	25.48

^*^X_2_ levels = 7, 8, and 9; ^**^X_6_ levels = 2, 2.5, and 3%; ^***^X_7_ levels = 1, 1.25, and 1.5%, w/v

The results of the analysis of variance of the model, ANOVA (Table 9) showed that transesterification capability in terms of FAME (%), lipase activity (U/g), and growth in terms of freeze-dried biomass models were significant, with P-values below 0.0001. The lack of fit of the three models was insignificant, with P-values higher than 0.05.

**Table 9:** Analysis of variance (ANOVA) for the parameters of response surface methodology fitted to a second-order polynomial equation

Source	DF	SS	MS	F-value	P-value > F
FAME					
Model	9	4219.81401	468.86822	111.542311	< 0.0001
Error (residual)	35	147.122537	4.2035011		
Lack of fit	5	14.6275369	2.9255074	0.66240403	0.654695
Pure error	30	132.495	4.4165		
Total	44	4366.93655			
Lipase					
Model	9	1084.37797	120.48644	102.815983	< 0.0001
Error (residual)	35	34.5100775	0.9860022		
Lack of fit	5	10.320746	2.0641492	2.01744367	0.104677
Pure error	30	30.6945253	1.0231508		
Total	44	1125.39324			
Biomass					
Model	9	989.507756	109.94531	111.506145	< 0.0001
Error (residual)	35	43.8302593	1.2522931		
Lack of fit	5	7.99489705	1.5989794	1.80912901	0.141196
Pure error	30	26.5151804	0.8838394		
Total	44	1024.01783			

Blotting the actual values obtained from the experiments against the predicted values deducted by the model (Additional file 1: Fig. S1) shows R^2^ values of 0.95, 0.91, and 0.92 for FAME (%), lipase activity, and growth, respectively.

The prediction formulas were simplified to second-order polynomial equations. The responses, FAME (Y_1_), Lipase (Y_2_), and growth (Y_3_), can be expressed in terms of the following regression equations:

$${\varvec{Y}}_{1} = \, 8.04{\varvec{X}}_{{\mathbf{2}}} - \, 71.70{\varvec{X}}_{{\mathbf{6}}} - \, 114.64{\varvec{X}}_{{\mathbf{7}}} + \, 0.42{\varvec{X}}_{{\mathbf{2}}}^{2} + \, 12.58{\varvec{X}}_{{\mathbf{6}}}^{2} - \, 0.04{\varvec{X}}_{{\mathbf{7}}}^{2} - \, 4.94{\varvec{X}}_{{\mathbf{2}}} {\varvec{X}}_{{\mathbf{6}}} - \, 0.35{\varvec{X}}_{{\mathbf{2}}} {\varvec{X}}_{{\mathbf{7}}} + \, 52.93{\varvec{X}}_{{\mathbf{6}}} {\varvec{X}}_{{\mathbf{7}}} + \, 138.02.$$

$${\varvec{Y}}_{2} = {2}.{\text{76X}}_{{2}} - {53}.{54}{\mathbf{X}}_{{\mathbf{6}}} - { 41}.{94}{\mathbf{X}}_{{\mathbf{7}}} + \, 0.{27}{\mathbf{X}}_{{\mathbf{2}}}^{{2}} + { 8}.{2}0{\mathbf{X}}_{{\mathbf{6}}}^{{2}} - { 5}.{17}{\mathbf{X}}_{{\mathbf{7}}}^{{2}} - { 1}.{83}{\mathbf{X}}_{{\mathbf{2}}} {\mathbf{X}}_{{\mathbf{6}}} - { 1}.{34}{\mathbf{X}}_{{\mathbf{2}}} {\mathbf{X}}_{{\mathbf{7}}} + { 28}.{91}{\mathbf{X}}_{{\mathbf{6}}} {\mathbf{X}}_{{\mathbf{7}}} + { 81}.{98}.$$

$${\varvec{Y}}_{3} = {23}.{44}{\mathbf{X}}_{{\mathbf{2}}} + { 54}.{29}{\mathbf{X}}_{{\mathbf{6}}} + { 9}.{1}0{\mathbf{X}}_{{\mathbf{7}}} + \, 0.0{5}{\mathbf{X}}_{{\mathbf{2}}}^{{2}} - { 5}.{64}{\mathbf{X}}_{{\mathbf{6}}}^{{2}} - { 4}.{37}{\mathbf{X}}_{{\mathbf{7}}}^{{2}} - { 5}.{81}{\mathbf{X}}_{{\mathbf{2}}} {\mathbf{X}}_{{\mathbf{6}}} - { 6}.0{5}{\mathbf{X}}_{{\mathbf{2}}} {\mathbf{X}}_{{\mathbf{7}}} + { 21}.{85}{\mathbf{X}}_{{\mathbf{6}}} {\mathbf{X}}_{{\mathbf{7}}} - { 164}.{8}0.$$

3D-surface plots were constructed to determine the optimum conditions, where the response was plotted on the Z axis against the two of the investigated factors were plotted on the X and Y axes. In contrast, the third factor was set to the optimal value. The model suggests that the optimum cultivation conditions to maximize *R. stolonifer* 1aNRC11 biomass transesterification capability are X_2_ = 7, X_6_ = 3%, and X_7_ = 1.5%, respectively w/v; lipase activity are X_2_ = 7, X_6_ = 3%, and X_7_ = 1.5%, w/v; and growth are X_2_ = 7, X_6_ = 3%, and X_7_ = 1.5%, w/v within the experimental space.

Expanding the model range where ranges were set to pH 3–11, the fishmeal concentration 0–6%, and KH_2_PO_4_ concentration 0–6%, suggests the optimum conditions to be X_2_ = 7.4, X_6_ = 2.62%, and X_7_ = 2.99% w/v. Both sets of the optimum conditions were tested for model validation (Table 10). The actual values of lipase activity and biomass were very close to the predicted values. The actual FAME values were relatively distant from the predicted values within the experimental and expanded ranges.

**Table 10:** Extra runs for the validation of CCD-suggested models

X_2_	X_6_	X_7_	FAME (%)		Lipase (U/g)		Growth (g/L)	
pH	(%, w/v)		Actual	Predicted	Actual	Predicted	Actual	Predicted
7.00	3.00	1.50	75.70	71.56	30.91	30.81	30.21	30.63
7.40	2.62	2.99	85.50	86.85	23.92	23.03	27.96	27.64

## Discussion

Whole-cell biocatalysts are often more potent than enzymes; in addition to the potential use of endogenous cofactors, whole-cell catalysis protects the catalytic protein from stress factors such as aeration, reactive substrates, or products [27]. Optimizing growth requirements to increase the catalytic capabilities of fungi is a common technique [28, 29]. The optimization of cultivation conditions to increase the catalytic capability of the fungal biomass has also been reported [30, 31]. *Rhizopus stolonifer* 1aNRC11 was isolated from the soil and selected because it showed a relatively high catalytic transesterification capability compared to other isolates; then, it was randomly mutated. The mutant that showed higher activity was chosen for this study [17]. During this study, the cultivation conditions of *R. stolonifer* were optimized to maximize the capabilities of the biomass to catalyze the transesterification of triglycerides considering the growth. The lipase capability of the produced biomass was also observed, as the correlation between lipase activity and transesterification capability of the fungal biomass has been reported in previous studies [17, 18], which might be due to the assumption that lipases displayed on the surface of the hyphae catalyze both reactions. The classical OFAT approach was followed to eliminate the agitation rate and the categorical factors, and a statistical approach was applied to optimize the continuous factors.

The agitation rate affects the rheology of the fermentation broth in submerged fermentation; hence the growth, biomass morphology, and composition of Mucorlean fungi are influenced by the agitation rate [32, 33]. Biomass produced under static conditions showed aerial growth and a fungal mat, while biomass grown in shaking flasks showed pellety growth that varies in size and hairiness. The significant improvement in the growth and biomass capability to catalyze triglycerides hydrolysis and transesterification was observed by comparing the static fermentation against the shaking flasks fermentation, which might be driven by the morphological difference between the biomass, which affects the surface area exposed to the substrate. Submerged fermentation at 200 rpm increased the produced biomass and its lipase and transesterification capability by 3.2, 4.8, and 3.1 folds, respectively, compared to static conditions. Therefore, further experiments were conducted at 200 rpm. Different oils, tested as activity inducers, had an insignificant effect on the growth.

A fungus should be able to produce extracellular or cell wall-integrated lipases to utilize triglycerides. Thus, vegetable oils induce lipase production by *Rhizopus sp.* [34]. Olive oil has been commonly used as a potent lipase inducer for fungi [35–37], which agrees with the present results where olive oil is superior to other inducers in triglycerides hydrolysis and transesterification. Some literature has mentioned that olive oil's relatively high oleic acid content might correlate with its potency as a lipase inducer [38, 39]. The insignificant difference in the produced biomass capability of hydrolysis and transesterification of triglycerides in culture media containing rapeseed oil and WFO might be due to the resemblance of both oils in the fatty acid profile. Olive oil was selected for further investigation.

Different N sources were tested, and statistically significant differences were detected among means of biomass concentrations, lipase activity, and transesterification capability of the produced biomass. *R. stolonifer* 1aNRC11 failed to grow in the medium containing urea as a sole nitrogen source which has been observed for other members of the genus Rhizopus [40, 41]. In the presence of CSL, a significant reduction in the growth and biomass capability to catalyze triglycerides hydrolysis and transesterification compared to the polypeptone containing medium. On the other hand, fishmeal was as efficient as polypeptone and had no significant differences in growth and biomass capability to catalyze triglyceride hydrolysis and transesterification. Fishmeal has been reported as a cheap, efficient nitrogen source for lipase production in several microorganisms, including *Rhizopus sp* [42–44]. Hence, fishmeal was selected to replace polypeptone in further investigations.

The selection of the factors for statistical screening relied mainly on the literature reviews and laboratory observations. The statistical screening of 9 factors using the Placket Burman design revealed the significant influence of most of the tested factors on one or more of the investigated responses. Some factors had negative main effects on the lipid modification capability of *R. stolonifer* 1aNRC11 biomass, such as glucose concentration and temperature. In contrast, others had a positive influence, such as MgSO_4_ concentration.

Glucose concentration negatively influenced the growth and catalytic activities of *R. stolonifer* 1aNRC11 in the investigated range, suggesting that the lower concentration (0.5%, w/v) is optimal compared to the higher concentrations; hence the low concentration was selected as an optimum concentration for further investigations. Some literature suggests that glucose has an inhibitory influence on the lipolytic activity of some fungi [45–47]. The C/N ratio, osmotic stress, and the tendency of the tested strain to utilize glucose over triglycerides might be potential physiological explanations for the observed adverse effect of the glucose concentration on growth and the catalytic capabilities of the produced biomass.

KH_2_PO_4_ role as a source of potassium and phosphate [48] might not be the only reason for the significant influence of KH_2_PO_4_, but also its buffering capacity [49, 50] This is supported by the finding that initial pH significantly affected biomass transesterification capability. The high significant main effect of KH_2_PO_4_ concentration made it a candidate for further optimization using RSM.

The influence of pH on the investigated responses was significant. However, the pH main effect value was positive for the transesterification capability and negative for the lipase activity, which could be because of the impact of pH on the composition of lipases [51] displayed on the surface of the biomass and/or the pH influence on the structural components and the folding of the active site of the lipases [52].

Fishmeal has been used as a nitrogen source to cultivate *Rhizopus* sp for several purposes, including lipase enzyme production [49, 53]. The data shows that fishmeal significantly influenced growth, the lipolytic and transesterification capabilities of the produced biomass as it is the sole N source in the culture medium in addition to the presence of some growth requirements in the fishmeal such as vitamins, minerals, and essential amino acids [54]. Fishmeal concentration was selected for further optimization using RSM based on the biomass transesterification capability’s significant main effect value (10.42).

Sulfur is an essential macronutrient for all living organisms with critical structural and metabolic roles [55]. Di-cations are substantial for fungal growth and as cofactors for several enzymes. Magnesium has been reported to enhance lipase production by fungi alone [56] or synergistically with other di-cations such as calcium [57]. The impact of MgSO_4_ concentration on growth and lipase and transesterification capability of the produced biomass could be explained in light of the reasons mentioned. MgSO_4_ concentration of 0.09 was selected as the optimum concentration for further investigations.

Inoculum size has been reported as a substantial variable affecting the growth of fungi and enzyme production [58]. However, inoculum size (1–3, 5 mm disks) significantly influenced the growth but had an insignificant influence on the produced biomass's catalytic activity. Hence inoculum size of 3 disks was selected for further investigations considering the positive main effect of the factor on the growth.

The incubation period is critical in fungal biosynthesis and autolysis rates of catalytic proteins [59], which explains the significant impact of the incubation period in the investigated range on the growth and biomass lipase activity of *R. stolonifer* 1aNRC11*.* However, the influence of the incubation period on transesterification capability was insignificant. The central value (3 days) was considered optimal to avoid the risk of lysis of catalytic proteins or biomass at higher incubation periods.

The temperature range of 25–30 °C significantly negatively affected lipase activity and transesterification capability of the produced biomass. However, it showed a significant positive impact on the growth of *R. stolonifer* 1aNRC11*.* On the other hand, olive oil concentration (10–30 gL^−1^) significantly influenced the tested criteria of *R. stolonifer* 1aNRC11*.*

According to the results of PB design, pH, fishmeal concentration, and KH_2_PO_4_ concentration had the highest significant main effects on transesterification capability and significantly affected the growth and lipase; therefore, they were selected for further optimization. Based on the transesterification capability results, other factors were fixed. Central values were chosen for insignificant factors, positive values for significant factors with a positive main effect, and negative values for significant factors with a negative main effect.

To determine the optimum levels of the selected significant factors for *R. stolonifer* 1aNRC11 biomass transesterification capability. For this purpose, the response surface methodology (RSM) was adopted, using a central composite design (CCD). The acceptability of the models was verified via the results of the analysis of variance, ANOVA. Transesterification capability in terms of FAME (%), lipase activity (U/g), and growth in terms of freeze-dried biomass models were significant, with P-values below 0.0001, and the lack of fit of the three models shows P-values higher than 0.05. Blotting the actual values obtained from the experiments against the predicted values deducted by the model supports the findings of the adequacy of the models, where R^2^ values were 0.95, 0.91, and 0.92 for FAME (%), lipase activity, and growth, respectively.

The transesterification capability model terms X_2_, X_6_, X_7_, X_6_^2^, X_2_X_6_, and X_6_X_7_ were highly significant with P-values less than 0.0001, while X_2_^2^, X_7_^2^, and X_2_X_7_ were insignificant. The lipase activity model terms X_2_, X_6_, X_7_, X_2_^2^, X_2_X_6,_ and X_6_X_7_ were significant, while X_2_^2^, X_7_^2^, and X_2_X_7_ were insignificant terms. On the other hand, only X_2_^2^ and X_7_^2^ were insignificant terms for the growth model.

The significant effect of X_2_X_6_ (pH and fishmeal concentrations interaction) on the transesterification and the lipase activities and growth might result from the availability of some micronutrients and/or growth promoters and the form of available nitrogen at different pH values [60–62]. The significant effect of the interaction between fishmeal and KH_2_PO_4_ concentrations (X_6_X_7_) could be because fishmeal is the sole N source which is required to build up the catalytic protein, and KH_2_PO_4_ is the primary source of P and K which has been reported to influence the lipase enzyme activity and composition of the enzyme [51]. The insignificance of pH and KH_2_PO_4_ concentration interaction (X_2_X_7_) on the transesterification and lipase activities was not expected in light of the buffering capacity of KH_2_PO_4_ and the initial medium pH; however, this might be due to the narrow range of the KH_2_PO_4_ in terms of buffering capacity and pH range investigated, which implies that the significant influence of KH_2_PO_4_ is because of its role as a source of potassium and phosphate. These results agree with the findings of Pimentel et al., but their results were developed using *Penicillium sp* [51, 63].

**Fig. 1:**
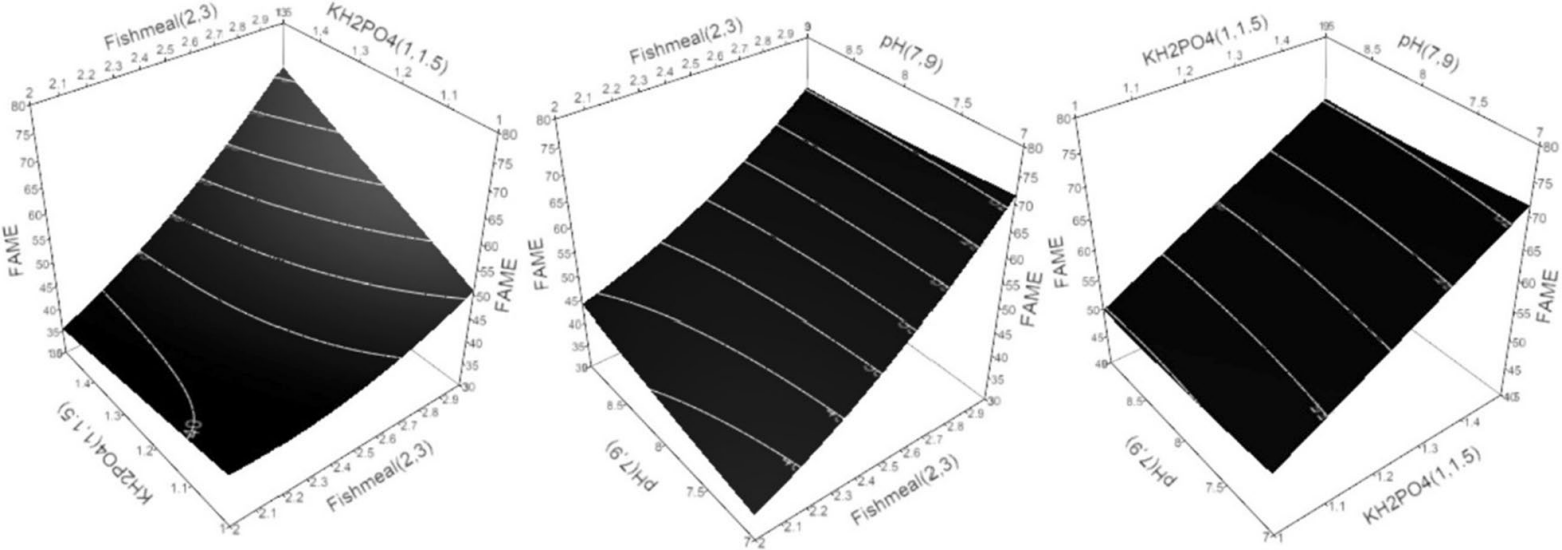
Three-dimensional response surface plots for biomass transesterification capabilities (FAME percentage) showing the interactive effects. Hold values are KH_2_PO_4_ = 1.5%, pH = 7, and Fishmeal = 3%

As shown in Fig. 1 linear increase in transesterification capability was observed when the fishmeal concentration and KH_2_PO_4_ concentration increased. The decline in the transesterification capability was associated with the rise in initial pH value. The model suggests that the optimum cultivation conditions for biomass transesterification capability within the experimental space are X_2_ = 7, X_6_ = 3%, and X_7_ = 1.5%, respectively w/v.

**Fig. 2:**
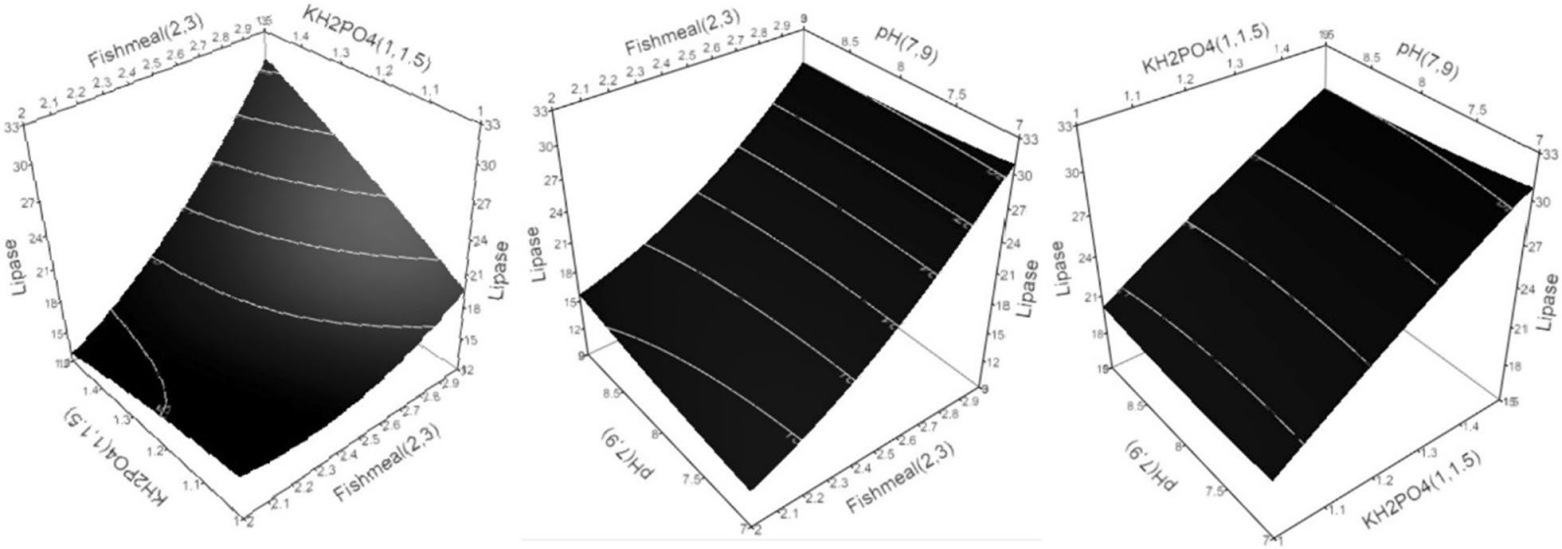
Three-dimensional response surface plots for biomass lipase activity showing the interactive effects. Hold values are KH_2_PO_4_ = 1.5%, pH = 7, and Fishmeal = 3%

Fishmeal concentration in the investigated range shows a linear correlation to the lipase activity, and the maximum lipase activity was achieved at a high fishmeal concentration (Fig. 2). An increase in lipase activity was associated with increased KH_2_PO_4_ concentration. A slight decline in lipolytic activity was observed with an increase in the initial pH value. The model suggests the optimum cultivation conditions for *R. stolonifer* 1aNRC11 biomass lipase activity within the experimental space to be X_2_ = 7, X_6_ = 3%, and X_7_ = 1.5%, w/v.

**Fig. 3:**
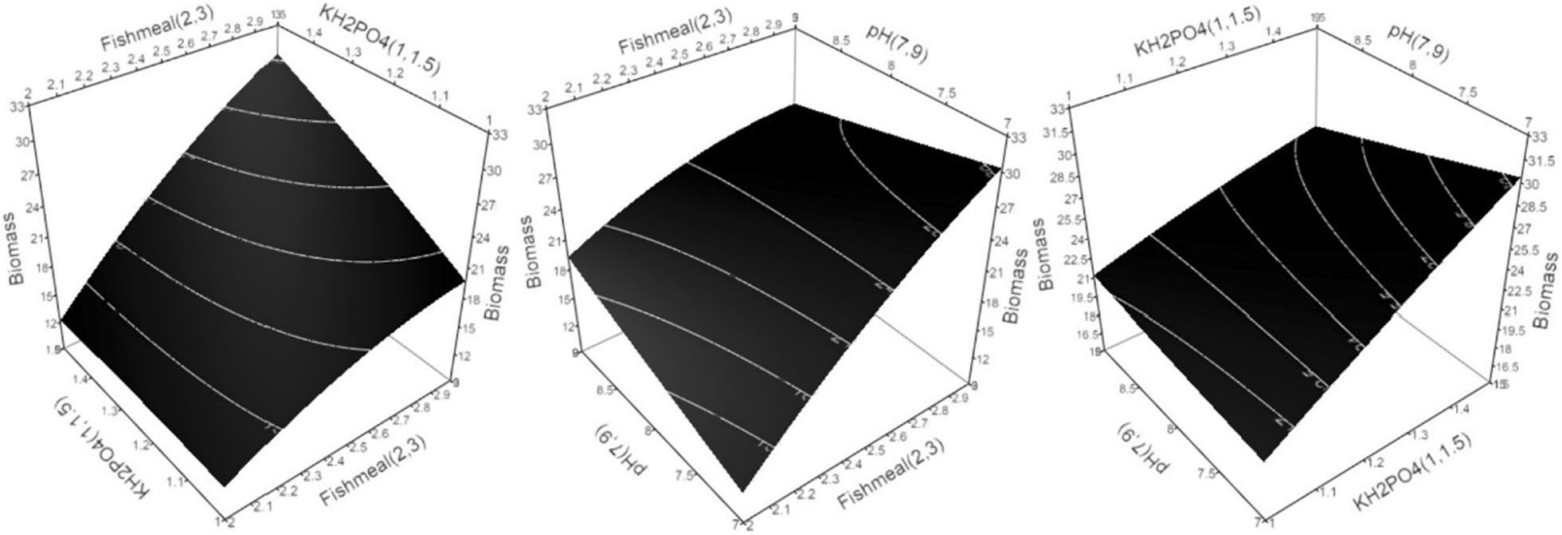
Three-dimensional response surface plots for biomass production (Growth) showing the interactive effects. Hold values are KH_2_PO_4_ = 1.5%, pH = 7, and Fishmeal = 3%

The growth shows a linear correlation with fishmeal and KH_2_PO_4_ concentrations, where increased fishmeal and/or KH_2_PO_4_ concentration leads to increased biomass production (Fig. 3). On the other hand, low initial pH values were associated with relatively high biomass production. The model suggests the optimum cultivation conditions for *R. stolonifer* 1aNRC11 growth within the experimental space to be X_2_ = 7, X_6_ = 3%, and X_7_ = 1.5%, w/v.

Determining optimum conditions for transesterification capabilities and lipase activity should always consider the produced biomass as an essential factor for selecting a sweet spot where maximum activity per gram is achieved along with high biomass production. The optimum conditions for the transesterification capability are identical to those required for the highest growth and lipase activity within the experimental space. The profile was set to optimize the variables to maximize transesterification capability and biomass production. The pH range was 3–11, the fishmeal concentration range was 0–6%, and the KH_2_PO_4_ concentration range was 0–6%. The model suggests the optimum conditions to be X2 = 7.4, X6 = 2.62%, and X7 = 2.99% w/v. Both sets of optimum conditions were tested for model validation (Table 7). The differences between the actual and the predicted FAME values were 1.35 and 4.14%, lipase 0.32 and 3.85, and biomass 1.37 and 1.16.

### Supplementary Information

**Additional file 1:** **Figure S1.** Actual Vs Predicted A: FAME B: Lipase C: Biomass showing R square.

## Data Availability

The datasets generated and analyzed during the current study and not included in this published article are available from the corresponding author upon reasonable request.
