# Supplementary figures and images for: Rhizopus stolonifer biomass catalytic transesterification capability: optimization of cultivation conditions
Source: Microb Cell Fact. 2023 Aug 14;22:154. doi: 10.1186/s12934-023-02141-y (PMC10424374; doi:10.1186/s12934-023-02141-y)

**Figure S1:** Actual Vs Predicted A: FAME B: Lipase C: Biomass showing R square.


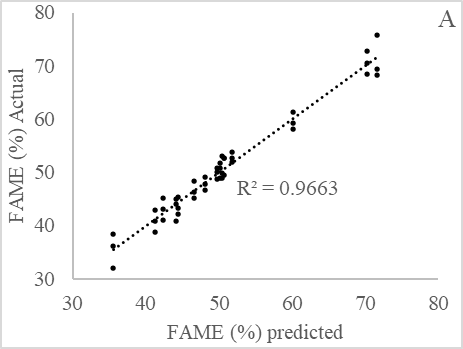

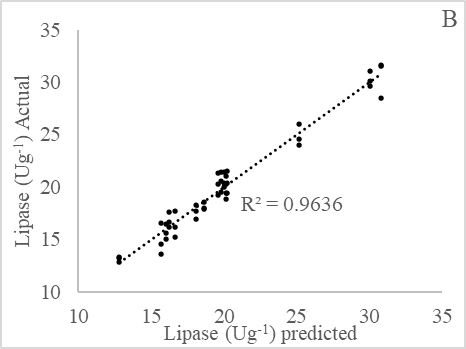

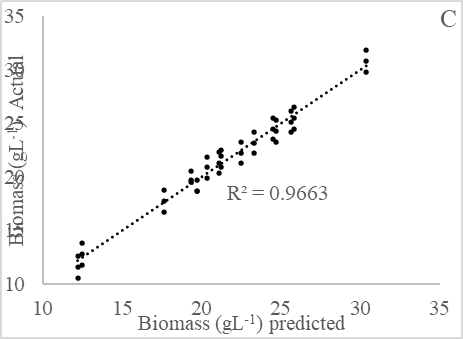

Supplement: Supplementary file 1 — Additional file 1: Figure S1. Actual Vs Predicted A: FAME B: Lipase C: Biomass showing R square. [file 12934_2023_2141_MOESM1_ESM.docx]
